# Beyond DNA Repair: Additional Functions of PARP-1 in Cancer

**DOI:** 10.3389/fonc.2013.00290

**Published:** 2013-11-27

**Authors:** Alice N. Weaver, Eddy S. Yang

**Affiliations:** ^1^Department of Radiation Oncology, School of Medicine, The University of Alabama at Birmingham, Birmingham, AL, USA; ^2^Department of Cell, Developmental, and Integrative Biology, School of Medicine, The University of Alabama at Birmingham, Birmingham, AL, USA; ^3^Department of Pharmacology and Toxicology, School of Medicine, The University of Alabama at Birmingham, Birmingham, AL, USA

**Keywords:** PARP-1, PARP inhibitors, NF-κB, genetic transcription, sex hormone signaling, ERK signaling, angiogenesis, mitotic spindle

## Abstract

Poly(ADP-ribose) polymerases (PARPs) are DNA-dependent nuclear enzymes that transfer negatively charged ADP-ribose moieties from cellular nicotinamide-adenine-dinucleotide (NAD^+^) to a variety of protein substrates, altering protein–protein and protein-DNA interactions. The most studied of these enzymes is poly(ADP-ribose) polymerase-1 (PARP-1), which is an excellent therapeutic target in cancer due to its pivotal role in the DNA damage response. Clinical studies have shown susceptibility to PARP inhibitors in DNA repair defective cancers with only mild adverse side effects. Interestingly, additional studies are emerging which demonstrate a role for this therapy in DNA repair proficient tumors through a variety of mechanisms. In this review, we will discuss additional functions of PARP-1 – including regulation of inflammatory mediators, cellular energetics and death pathways, gene transcription, sex hormone- and ERK-mediated signaling, and mitosis – and the role these PARP-1-mediated processes play in oncogenesis, cancer progression, and the development of therapeutic resistance. As PARP-1 can act in both a pro- and anti-tumor manner depending on the context, it is important to consider the global effects of this protein in determining when, and how, to best use PARP inhibitors in anticancer therapy.

## Introduction

Poly(ADP-ribose) polymerase-1 (PARP-1) is a nuclear enzyme which binds DNA via two zinc finger motifs and transfers chains of ADP-ribosyl moieties (PARs) from nicotinamide-adenine-dinucleotide (NAD^+^) to chromatin-associated acceptor proteins, including PARP-1 itself. This post-translational modification plays an important role in promoting DNA repair by releasing PARP-1 from DNA and allowing for recruitment of proteins involved in both base excisional repair (BER) and homologous recombination (HR) (1). Accordingly, PARP-1 is an attractive anticancer target, and poly(ADP-ribose) polymerase (PARP) inhibitors have been identified as chemo- and radiation-sensitizing agents in an array of cancers (2–5), including our report on the sensitization of head and neck cancer to radiotherapy following PARP inhibition (6). Perhaps the most well-known tumoricidal effects of PARP inhibitors are in BRCA-mutated cancers, which harbor DNA repair defects and become dependent on PARP-1-mediated repair for survival. Two landmark studies (7, 8) found inhibition of PARP-1 in cells containing BRCA mutations resulted in the generation of chromatid breaks, G2 cell cycle arrest, and enhancement of apoptosis, results which have been confirmed in early phase clinical trials (9, 10).

**Figure 1:**
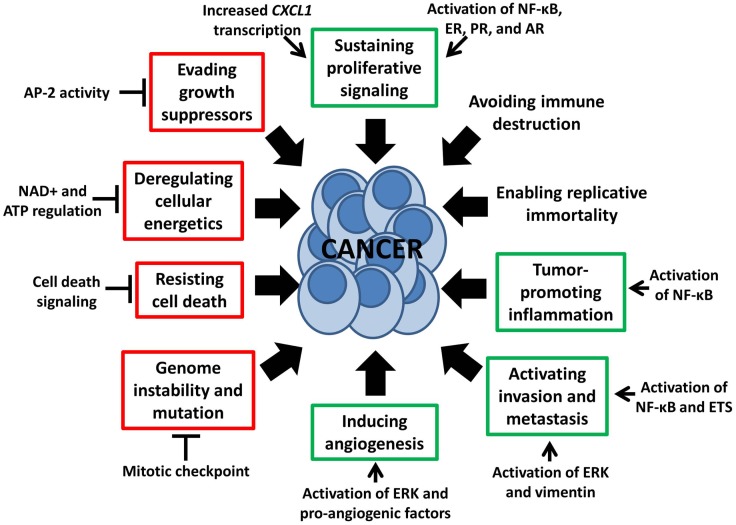
**Non-DNA repair functions of PARP-1 influence the “hallmarks of cancer” (18)**. This schematic depicts multiple PARP-1-mediated processes which either stimulate or inhibit six of the eight “hallmarks of cancer,” as indicated by green and red boxes respectively. These hallmarks, proposed by Hanahan and Weinberg, are malignant characteristics that provide a framework for understanding the biology of cancer.

Interestingly, recent studies also show potential efficacy of PARP inhibition in sporadic tumors lacking DNA repair defects. A clinical study of the PARP inhibitor olaparib in women with heavily pretreated high-grade serous ovarian cancer without germline BRCA1/2 mutations resulted in objective responses in 11/46 (24%) (11), indicating there may be additional determinants of sensitivity to PARP inhibition. Pre-clinical studies have identified susceptibility to PARP inhibition alone in HR-proficient HER2-positive breast cancer, pancreatic cancer, prostate cancer, Ewing’s sarcoma, small cell lung carcinoma, and neuroblastoma, among others (12–17). These reports demonstrate the existence of non-DNA repair functions of PARP-1 that may be targetable for cancer treatment. It is thus becoming increasingly apparent that a number of PARP-1-mediated cellular processes influence characteristics of tumor development, progression, and treatment response, including several of the eight “hallmarks of cancer” proposed by Hanahan and Weinberg (18) (Figure 1). In this review, we will discuss cancer-related functions of PARP-1 – including regulation of inflammatory mediators through NF-κB, cell death and energetics, ERK-mediated tumor progression and invasion, mitosis, gene transcription, and sex hormone signaling – and examples of how these functions may be exploited to expand the patient population potentially benefiting from treatment with PARP inhibitors.

## NF-κB-Mediated Tumor-Promoting Inflammation

In multiple cancers, including breast, prostate, and head and neck among others, the NF-κB signaling pathway undergoes a loss of regulation resulting in constitutive activation (19). Briefly, NF-κB is a family of transcription factors including RelA/p65, RelB, c-Rel, p50, and p52, which exist as homo- and hetero-dimers. DNA-binding affinity and DNA sequence specificity is dependent on the composition of the dimer. Inhibitory proteins bind NF-κB dimers and sequester them in the cytosol in the absence of a stimulus; pathway activation causes proteasomal degradation of inhibitors, allowing the dimer to translocate to the nucleus and activate pro-inflammatory transcription programs. Although NF-κB signaling mediates the acute immune response responsible for targeting and eliminating cancerous cells, chronic inflammation mediated by this “hallmark” pathway can lead to the malignant phenotype (Figure 1), facilitating escape from immune surveillance, cancer survival, metastasis, and angiogenesis (20).

**Figure 2:**
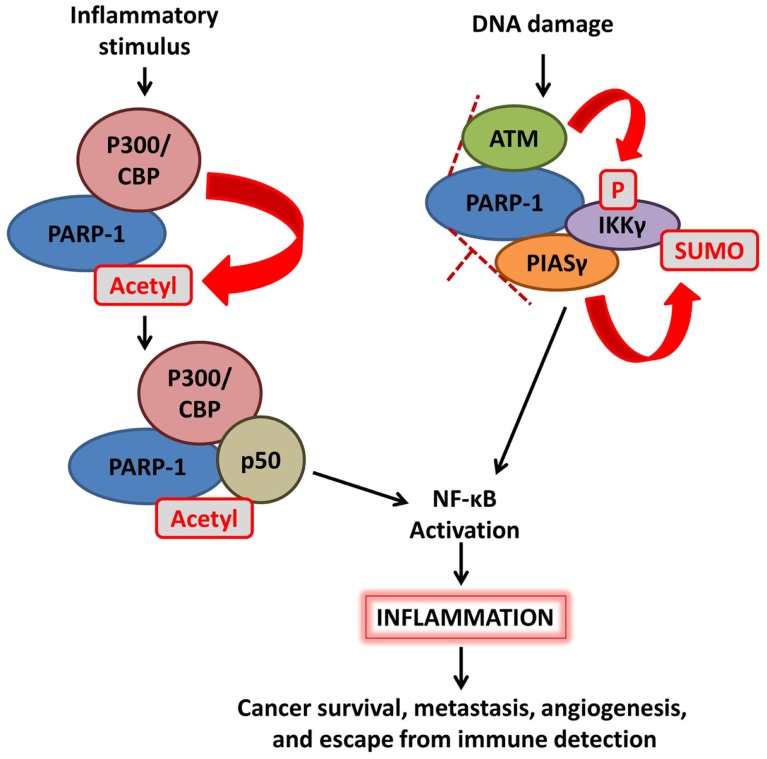
**Poly(ADP-ribose) polymerase-1 mediates activation of NF-κB signaling**. (Left) inflammatory stimulation triggers p300/CBP acetylation of PARP-1, enhancing the interaction between p50 and PARP-1 as well as the p50–p300 interaction; this ultimately leads to activation of NF-κB. (Right) DNA damage detection promotes the formation of a complex including PARP-1, ATM, PIASγ, and IKKγ (NEMO); chains of PAR on PARP-1 provide a structure upon which PIASγ SUMOylates IKKγ, leading to NF-κB activation.

Activation of NF-κB can be regulated by PARP-1 via multiple mechanisms (Figure 2). First, PARP-1 directly interacts with histone acetyl-transferases p300 and CREB-binding protein (CBP) to synergistically co-activate NF-κB-dependent gene expression. In response to inflammatory stimuli, p300/CBP acetylates PARP-1 at specific lysine residues. This modification is necessary for PARP-1-p50 interaction, enhancement of p300–p50 interaction, and co-activation of NF-κB-mediated transcription programs (21, 22). Co-activation is negatively regulated by the activity of class I histone deacetylases (HDACs) (22) and SUMO1/3-mediated SUMOylation of the automodification domain of PARP-1 (23). Second, enzymatic activation of PARP-1 variably affects NF-κB, with outcomes dependent on the identity of the PAR acceptor protein. AutoPARylation of PARP-1 following detection of DNA strand breaks promotes the formation of a “signalosome” containing IKKγ (NEMO), the regulatory subunit of a NF-κB inhibitory complex, along with PIASγ, and ATM. Chains of PAR on activated PARP-1 provide the scaffold needed for SUMOylation of IKKγ by the PIASγ PAR binding motif, leading to activation of IKK and NF-κB (24). The effects of PARylation on NF-κB itself are less clear, with different sources reporting decreased, increased, or unaffected DNA-binding activity (25–27). Taken together, these studies demonstrate a strong role for PARP-1 in regulating NF-κB activity.

The interaction between PARP-1 and the NF-κB pathway promotes production of pro-inflammatory cytokines such as TNFα, IL-6, INFγ, E-selectin, and ICAM-1, as well as expression of nitric oxide synthase (28–30); PARP inhibition has been shown to attenuate upregulation of these factors in response to inflammatory stimuli (28, 29). Furthermore, PARP inhibition may also prevent inflammation-associated adverse side effects of traditional chemotherapeutics (31), supporting the use of PARP inhibitors in multidrug regimens. Loss of PARP-1 activity not only decreases pro-tumor inflammation, but also inhibits two related hallmarks of cancer through anti-inflammatory mechanisms: proliferative signaling (32) and metastasis (33, 34) (Figure 1).

Recently, we discovered an unexpected sensitivity to PARP inhibition in DNA repair proficient HER2-positive breast cancer cells through attenuation of NF-κB-mediated signaling (13). HER2 over-expressing cancers have activated NF-κB, which acts to block apoptosis and possibly mediate resistance to HER2-targeted drugs (35). In HER2-positive breast cancer cells, treatment with PARP inhibitor significantly reduced the expression of NF-κB activator IKKα and phosphorylated p65 while increasing inhibitory IkBα. These events resulted in decreased NF-κB transcriptional activity in HER2-positive, but not HER2-negative, breast cancer cells (13). Furthermore, overexpression of HER2 alone was sufficient to confer sensitivity to PARP inhibitor, suggesting synthetic lethality with PARP inhibition in tumors that are oncogene-addicted to HER2 signaling through NF-κB. This study represents a specific application of PARP-1-regulated NF-κB signaling to cancer therapy, one that may soon be expanded into a clinical trial.

## Cellular Energetics and Cell Death

Cancer cells are characterized by excessive proliferation, impaired cell death signaling, and deregulated metabolism (Figure 1). These features are often mediated by altered mitochondrial activity coupled with inactivation of apoptotic signaling through decreased expression of pro-apoptotic factors like p53 or overexpression of anti-apoptotic factors like Bcl-x. Integrity of regulatory pathways for cell death and metabolism is important for response to many cancer treatment modalities, as well as in cancer imaging and diagnostics. Cellular energetics and death signaling are heavily regulated by PARP-1, allowing activity of this protein to serve as a switch between cell fates and to affect both tumor proliferation and therapeutic response.

**Figure 3:**
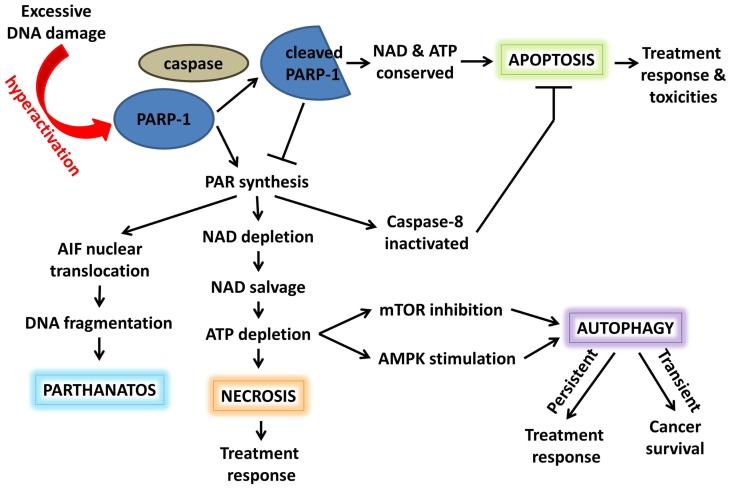
**Poly(ADP-ribose) polymerase-1 acts as a switch between cell fates**. Hyperactivation of PARP-1 and PAR synthesis depletes NAD and, subsequently, ATP. Elevated PAR can promote necrosis, autophagy, or AIF-induced parthanatos. In addition, PARylation inactivates caspase-8, inhibiting apoptotic signaling. Alternatively, activated caspases can cleave PARP-1; the resulting cleavage product inhibits uncleaved PARP-1, conserving NAD/ATP, and promoting apoptosis. These cell death pathways play a role in both cancer survival and response to anticancer therapy.

In response to damage stimuli, activated PARP-1 acts early in the apoptosis initiation pathway to stabilize p53 and facilitate its function (36). If damage is excessive, high levels of PAR synthesis by PARP-1 deplete its NAD^+^ substrate; additional interactions between PARP-1 and NMNAT-1, a NAD^+^ synthase, and SIRT1, a NAD^+^-dependent protein deacetylase, further contribute to PARP-1 as a controller of NAD^+^ availability and, thus, NAD-dependent metabolic reactions. ATP-dependent NAD^+^ salvage saps cellular ATP stores, resulting in energy deprivation and, eventually, energy crisis-induced necrosis (Figure 3). Furthermore, PARP-1-mediated PARylation may inactivate caspase-8 and reduce caspase-mediated apoptotic signaling (37). Hyperactivation of PARP-1 and accumulation of PAR can also cause translocation of PAR to the cytosol, where it interacts with the outer mitochondrial surface. Here it binds apoptosis inducing factor (AIF) and induces its release and translocation to the nucleus, ultimately resulting in large-scale DNA fragmentation and a novel PARP-1-dependent cell death mechanism known as “parthanatos” (38). To prevent these events, activated caspases cleave PARP-1 into two fragments: an 89-kDa C-terminal fragment with low levels of catalytic activity and a 24-kDa N-terminal peptide which inhibits the catalytic activity of uncleaved nuclear PARP-1. Conservation of NAD^+^ and, thus, ATP allows the cell to undergo programed cell death (39–41). Accordingly, inhibition of PARP-1 preserves ATP levels, improves antioxidant status, and normalizes anti-apoptotic Bcl-x levels in the kidney following chemotherapy-induced injury (42, 43).

Poly(ADP-ribose) polymerase-1 also regulates the classical necroptotic pathway mediated by the death promoting MAP kinase, c-Jun N-terminal kinase (JNK). This signaling network is activated in many cancers and has been implicated as a driver of both tumor development and treatment response (44, 45). PARP-1 downregulates MAP kinase phosphatase MKP-1 expression and inhibits the survival kinase Akt, both of which activate JNK (46, 47), suggesting potential benefit for PARP inhibition in tumors with elevated JNK activity. JNK1 mediates phosphorylation and sustained activation of PARP-1, creating a feed-forward regulatory loop (48). In conjunction, PARP-1-induced depletion of ATP stimulates AMP-activated protein kinase (AMPK) while inhibiting mTOR to promote autophagy, yet another cell death pathway important in cancer survival and treatment response (49). Pharmacologic inhibition of PARP-1 promotes Akt activity and mTOR signaling resulting in decreased cell death (50), although these results are contradicted by a recent report showing PHLPP1-mediated downregulation of Akt activity and increased cell death following PARP inhibition (51).

Clinically, targeting the role of PARP-1 in cell death pathways appears to be complex. PARP-1 inhibition may reduce PAR-mediated inactivation of caspase-8, sensitizing cancer cells to tumor necrosis factor-related apoptosis-induced ligand (TRAIL) therapy (37). Additionally, inhibition of PARP-1 prevented cisplatin- and methotrexate-induced ATP depletion and nephrotoxicity (42, 43), as well as imatinib (Gleevec)-induced JNK activation and cardiotoxicity (52), without significantly affecting the anticancer activity of these agents. However, activation of the Akt survival pathway may counteract the cytotoxic effects of PARP inhibition and cause resistance to therapy (47), suggesting Akt pathway inhibition may enhance PARP inhibition in anti-tumor therapy. Despite these complexities, the influence of PARP-1 on metabolic co-factors and cell death signaling is significant, and further studies examining the role of PARP inhibition in manipulating these processes is warranted.

## ERK-Mediated Angiogenesis and Metastasis

In addition to the JNK-mediated signaling described previously, a second family of MAP kinases known as extracellular signal-regulated kinases or ERKs is involved not only in cell death determination but also in tumor progression, angiogenesis, and metastasis. ERK activation is pivotal in cancer cell survival through upregulation of anti-apoptotic proteins and inhibition of caspase activity (53). Inhibition of this pathway by targeting ERK or MEK, which is immediately upstream of ERK in signaling, has been associated with suppression of ovarian tumor growth (54), reduced metastatic potential of melanoma cells (55), and increased sensitivity to cytotoxic agents (56). Recent studies indicate an important role for PARP-1 in promoting ERK signaling.

Poly(ADP-ribose) polymerase-1 is activated and autoPARylated by a direct interaction with phosphorylated ERK2 (pERK2), resulting in enhanced pERK2-catalyzed phosphorylation of target transcription factors and increased gene expression (57). Furthermore, PARP inhibition causes loss of ERK2 stimulation by decreasing the activity of critical pro-angiogenic factors including vascular endothelial growth factor (VEGF), transmembrane signaling protein syndecan-4 (SDC-4), platelet/endothelial cell adhesion molecule (PECAM1/CD31), and hypoxia inducible factor (HIF). This ultimately results in reduced angiogenesis and inflammation (58–62). The effects of PARP-1 on ERK signaling are further enhanced by PARP-1-mediated transcription of vimentin, an intermediary angiogenic filament upregulated in tumor vasculature and pivotal for the endothelial-to-mesenchymal transition characteristic of metastasis (63). Pharmacologic inhibition of PARP reverted this transition, correlating with a reduction in the number and size of metastatic melanoma foci in a mouse model (63).

Collectively, these studies indicate PARP-1 directly fosters ERK signaling in addition to mediating separate but parallel signaling pathways reinforcing the same end result of increased angiogenesis and metastasis, two tumor-promoting features (Figure 1). As such, PARP inhibition may be effective in blocking the ERK signaling network or increasing activity of ERK/MEK inhibitors, agents already shown to be efficacious in acute myeloid leukemia, multiple myeloma, melanoma, colorectal, breast, lung, and pancreatic cancers (64–68). Furthermore, selective ERK inhibition induces tumor regression in MEK inhibitor-resistant models (67), raising the question of whether PARP inhibition could be similarly effective in either MEK or ERK-resistant tumors due to its proximity in the signaling pathway. As MEK, ERK, and PARP inhibitors have only recently entered early phase clinical trials, it will be some time before we know which patients benefit most from these drugs, either alone or in combination, but their interaction warrants further investigation.

## Mitotic Regulation

The high proliferation rate of cancer cells is a result not only of decreased cell death but also of improperly regulated cell cycling, allowing evasion of growth suppressing signals. Although multiple cell cycle checkpoints can be impaired in cancer, the mitotic or spindle assembly checkpoint is of great importance both in tumorigenesis and as an anticancer target. This point of regulation, which is responsible for ensuring appropriate chromosome segregation, is required for cell viability. Cells with a weakened mitotic checkpoint are capable of survival but do not maintain proper chromosome segregation, resulting in genomic instability and aneuploidy. These are common features of tumor cells and may even act as drivers in cancer development (Figure 1). PARP-1 can act on many mediators of cell cycle progression through its effects on gene expression (68), which will be detailed in a later section. However, direct regulation of the mitotic checkpoint by PARP-1 is another important factor that may be targetable in cancer treatment.

Recent reports suggest multiple roles for PARP-1 in the structural machinery of mitosis. First, PAR, which is primarily synthesized by PARP-1, is required for assembly and function of the bipolar spindle (69). In addition, PARP-1 both localizes to and PARylates proteins at centromeres and centrosomes during mitosis (70, 71). PARP-1 also mediates PARylation of p53, which is responsible for regulating centrosome duplication and monitoring chromosomal stability (71). Loss of PARP-1activity is associated with mislocalization of centromeric and centrosomal proteins, resulting in incomplete synapsis of homologous chromosomes, defective chromatin modifications, and failure to maintain metaphase arrest, indicating loss of mitotic checkpoint integrity (71, 72). Similarly, inhibition of PARP-1 is associated with genomic instability characterized by reduced stringency of mitotic checkpoints, centrosome hyperamplification, and chromosomal aneuploidy, the most common characteristic of solid tumors (71, 73, 74).

Furthermore, PARP-1 has been shown to interact with the E3 ubiquitin ligase, CHFR, a tumor suppressor with an important role in the early mitotic checkpoint. Binding of these two proteins results in degradation of PARP-1 and cell cycle arrest in prophase, an effect stimulated by the microtubule inhibitor docetaxel resulting in resistance to this drug in CHFR-over-expressing cancer cells. Concomitant use of a PARP inhibitor with docetaxel significantly increased apoptosis in these cells, suggesting a role for PARP inhibition in sensitizing cancers with high CHFR activity to microtubule inhibitors (75).

## Gene Transcription

**Figure 4:**
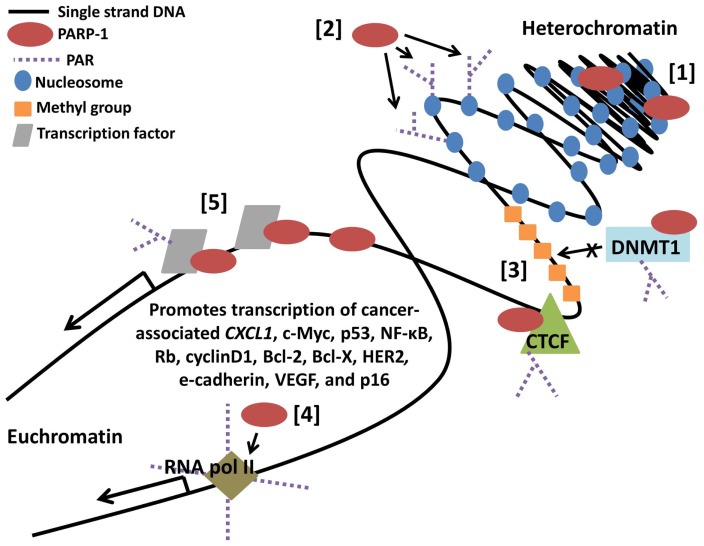
**Poly(ADP-ribose) polymerase-1-regulates gene transcription through multiple mechanisms**. [1] PARP-1 binds neighboring nucleosomes resulting in chromatin compaction. [2] PARP-1 PARylation of core histones mediates chromatin relaxation. [3] PARP-1 promotes hypomethylation of DNA by enhancing the chromatin insulator activity of CCCTC-binding factor (CTCF) while inhibiting methyltransferase activity of DNMT1. [4] PARP-1 promotes loading and retention of RNA polymerase II at active promoters. [5] PARP-1 binds regulatory DNA sequences and transcription factors, PARylates transcription factors, and recruits additional regulatory binding proteins in a target gene specific manner.

The clinical characteristics of cancer, including growth, metastatic potential, and response to treatment, are greatly influenced by dysregulation of gene transcription. Gene expression profiles are currently being utilized as tumor biomarkers, indicators of treatment sensitivity or resistance, and prognostic predictors. In the future, there may even be a role for therapeutic agents that reactivate a silenced tumor suppressor or silence an activated oncogene. In total, 3.5% of the transcriptome is regulated by PARP-1 with 60–70% positively regulated (76), including genes involved in tumor promotion such as *JUND, MDM2, HGF, FLT1* (VEGFR1), *EGFR, HIF2A* (EPAS1), *SPP1* (OPN), *MMP28, ANGPT2*, and *PDGF* (77). As discussed below and shown in Figure 4, this regulation can occur broadly through interactions with nucleosomes and modification of chromatin, can be gene specific through interactions with promoters and binding factors, or can result as a combination of the two, as binding of PARP-1 to nucleosomes mediates its localization to specific target gene promoters (78, 79).

### Chromatin structure

One mechanism by which PARP-1 alters gene expression is through regulation of chromatin structure and, thus, DNA accessibility. Simultaneous binding of multiple neighboring nucleosomes by PARP-1 compacts chromatin into a supranucleosomal structure, repressing gene transcription (79). This structural change is further stimulated by histone deacetylation mediated by a complex consisting of PARP-1, ATP-dependent helicase Brg1 (SmarcA4), and HDACs (80). Conversely, PARylation of core histones promotes charge repulsion-induced relaxation of chromatin and recruitment of transcription machinery (81–83). PARP-1-mediated PARylation also results in disassociation of linker histone H1, a repressor of RNA polymerase II-mediated transcription; accordingly, higher proportions of PARP-1:H1 indicate active promoters (84), suggesting potential utility of PARP-1 as a biomarker for actively transcribed genes. Although these outcomes can be separated by PARP-1 activity (protein binding versus enzymatic function), pharmacologic inhibition of PARP affect both actions, indicating manipulation of chromatin accessibility through PARP-1 is not currently an option for cancer therapy.

### Methylation patterns

Along with chromatin structure, methylation patterns also play a large role in determining DNA accessibility. Alterations in DNA methylation are commonly found in many cancers and serve as a functional equivalent to a gene mutation in the process of tumorigenesis. Inhibition of PARP-1 is associated with transcriptional silencing through accumulation of DNA methylation and CpG island hypermethylation throughout the genome (85). This effect may be mediated by dimerization of PARP-1 with CCCTC-binding factor (CTCF), a chromatin insulator which binds to hypomethylated DNA regions. As the CTCF-PARP-1 interaction is PAR-dependent, decreased PAR following PARP inhibition abrogates this function (86, 87). Loss of CTCF-PARP-1 complex activity results in transcriptional silencing of multiple loci including tumor suppressors *CDKN2A-INK4* (p16), *CDH1* (e-cadherin), and *P19ARF* (88, 89).

Poly(ADP-ribose) polymerase-1 can also hinder DNA methylation by dimerization with DNA (cytosine-5-)-methyltransferase 1 (DNMT1), a methyltransferase found overexpressed in gastrointestinal tract carcinomas, resulting in inhibition of its methyltransferase activity (85, 90). In contrast, PARP-1 binding and PARylation of the *Dnmt1* promoter actually enhances its transcription by preventing methylation-induced silencing (91). The reduced catalytic efficiency of PARylated DNMT1 may come as a result of negatively charged PARylated PARP-1 out-competing DNA for binding with DNMT1 (92). Interestingly, PARP-1-DNMT1 can form a ternary complex with CTCF at unmethylated CTCF-target sites in a PAR-dependent manner. Loss of PAR from this complex causes dissociation of PARP-1 and CTCF, allowing the still-bound DNMT1 to methylate the site and inhibit transcription (92).

Although some specific tumor suppressors are mentioned above as being affected by PARP-1-mediated chromatin insulation, the activity of PARP-1 in regulating DNA methylation patterns at specific genes or genic regions is largely unknown. As such, it is difficult to predict the effect of PARP inhibition on cancer growth and progression through this mechanism. However, with the advent of genomic profiling, it has recently become possible to identify methylation changes specific to certain cancer subtypes. Anticancer agents with epigenetic modifying activity, such as DNA methyltransferase inhibitors, are being investigated in these cancers and show promising results, especially in hematologic malignancies (93). The effect of PARP inhibition on epimutations has not been studied, but the reports described above suggest PARP inhibitors could have similar applicability.

### RNA polymerase II activity

Poly(ADP-ribose) polymerase-1 can also promote transcription in a more sequence-specific manner by positively regulating RNA polymerase II activity at active promoters. This occurs through: (1) PARylation-induced exclusion of histone demethylase KDM5B, maintaining levels of activating histone mark K3K4me3 (82), (2) PARylation-induced dissociation of the DEK repressor, promoting loading of the RNA polymerase II mediator complex (94), and (3) creation of a PAR scaffold for retention of RNA polymerase II (95). Surprisingly, a recent report showed that inhibition of PARP-1 enzymatic activity was associated with increased H3K4me3, resulting in upregulation of sodium iodide symporter transcription and elevated radio-iodine uptake in thyroid cancer cell lines (96). This contradictory work may result from target gene specific functions of PARP-1, as the previously cited studies were focused on genes known to be positively regulated by PARP-1. However, it does illustrate the need for greater understanding of PARP-1 involvement at active gene promoters, as well as the potential for manipulating PARP-1-mediated transcription to enhance efficacy of cancer therapy.

### DNA and transcription factor binding

Gene expression can be further regulated by direct interactions between PARP-1 and DNA elements or binding factors. PARP-1 acts as a promoter-specific switch at target genes, facilitating the release of inhibitory co-regulators and recruitment of stimulatory co-regulators (97, 98). PARP-1 binding of the NF-κB immediate upstream region (IUR) element activates transcription of *CXCL1*, which encodes melanoma growth stimulatory activity protein and is overexpressed in the progression of malignant melanoma (99). Binding of PARP-1 to the transcription factor E2F-1 increases E2F-1 promoter activity and expression of the E2F-1-responsive oncogene *Myc* (c-Myc) (100). PARP-1 expression and activity are also required for cancer cell invasion (Figure 1) mediated by ETS transcription factors – whose fusion products drive Ewing’s sarcoma, acute myeloid leukemia, and prostate cancer – and the Ewing’s sarcoma fusion protein EWS-FLI (14, 15). While PARP-1 interaction with these factors promotes pro-tumor signaling, other interactions have the opposite effect. PARP-1 suppresses self-inhibition of AP-2, a transcription factor that negatively regulates cell cycle and proliferation (101). Increased AP-2 expression suppresses cancer cell growth (102) and may inhibit *ras* oncogene-mediated transformation (101), effects likely diminished by PARP inhibition (Figure 1). PARP-1 has also been shown to bind the inhibitory element of COX-2, which mediates inflammation and promotes VEGF-mediated pro-angiogenesis pathways activated in cancer cells (103, 104).

Instances of PARP-1-mediated enzymatic activity affecting specific transcription factors or genes often translate to a clear role for PARP-1 inhibitors as anticancer agents, even in monotherapy. For example, ETS-positive prostate tumors and EWS-FLI-positive Ewing’s sarcomas are highly sensitive to PARP inhibitors (14, 15). However, PARP-1 has multiple and diverse functions involving both PARylation activity and DNA-binding capability. Enzymatic inhibition, which decreases PARP-1 self PARylation, actually increases DNA binding and may be detrimental in some cancers, such as the malignant melanoma example given above. A greater understanding of the relative effects of PARP-1 on transcriptional activity is needed in order to select tumors with a molecular profile conducive to pharmacologic inhibition through this mechanism.

## Sex Hormone Signaling

Sex hormones have been implicated in development, progression, and treatment sensitivity of prostate, breast, gynecologic, and colon cancers. Sex steroid effects are mediated through their receptors, which act as transcription factors in steroid-responsive tissues. Any of the multiple levels of regulation controlling these signaling pathways can become impaired, leading to abnormal proliferative responses characteristic of cancer progression (Figure 1). Similar to PARP-1-mediated regulation of transcription factor activity, PARP-1 plays a role in regulating three of the sex hormone receptors most commonly linked to cancer: estrogen receptor (ER), progesterone receptor (PR), and androgen receptor (AR).

Approximately 80% of breast carcinomas are positive for ER, identifying ER-targeted therapies as excellent, although not un-failable, treatment options in these cancers (105). PARP-1 interacts with the ERa isoform both directly and through estradiol-induced PARylation to enhance binding of ERa and other activating factors to target gene promoters (106, 107), suggesting PARP inhibition may enhance the activity of ER-targeted agents. A similar interaction occurs between PARP-1 and PR: PARP-1 binding of PR, as well as hormone-activated CDK2-induced PR PARylation, acts to stimulate cancer cell proliferation (108). PARP-1 regulation of PR activity is of great interest in endometrioid carcinomas specifically, as expression of PARP-1 and PR is positively correlated at each pathologic stage of this cancer (109). However, the effects of PARP inhibition in endometrial cancer have yet to be determined.

Recently, a report detailing the strong interaction between PARP-1 and AR has generated much excitement over the potential for PARP inhibitors in prostate cancer treatment. Human prostatic adenocarcinoma, a cancer highly resistant to standard therapies, is reliant on AR activity for growth and survival. Accordingly, AR-targeted therapies are the primary treatment for these patients. Unfortunately, there are multiple mechanisms for AR reactivation leading to tumor recurrence, a lethal phenotype known as castration-resistant prostate cancer. PARP-1 enzymatic activity, which is significantly upregulated in castration-resistant prostate cancer, promotes both AR chromatin binding and transcription factor functions. Although PARP-1 does localize with AR to regulatory sites of AR-target genes, the two proteins appear to be members of separate complexes at these loci. Inhibition of PARP-1 *in vivo*: (1) depletes both PARP-1 and AR at target genes, (2) significantly reduces expression of target genes, including pro-tumorigenic *ets* genes referenced previously, (3) sensitizes both castration-resistant and castration-sensitive prostate cancer cells to genotoxic insult and androgen depletion, (4) enhances the anti-tumor effects of anti-androgen therapy, and (5) delays onset of resistance to anti-androgen therapy. *Ex vivo* studies of castration resistance prostate tumors displayed a significant anti-tumor response to both veliparib and olaparib, two well-known PARP inhibitors, that correlates with reduced AR activity (110). These results suggest PARP inhibitors have the potential to significantly enhance existing prostate cancer therapy and improve outcomes for patients with castration-resistant tumors.

## Promise and Challenges

Poly(ADP-ribose) polymerase inhibitors are exciting new drugs that are easily delivered, can be highly efficacious, and are associated with few side effects. Mild nausea is commonly reported, with rare instances of more serious symptoms such as temporary cognitive deficits and myelosuppression. While ongoing clinical trials are focused on exploiting the role of PARP-1 in DNA repair, we have identified in this review multiple targetable functions of PARP-1 that are not dependent on HR defects (Figures 1–4; Table 1). One of the challenges in broadening the use of PARP inhibitors in anticancer therapy is more efficient identification of patients who may respond to these drugs. Some ongoing clinical trials include analysis of protein expression – including HR proteins, NF-κB, and PARP-1 itself – in relation to clinical response in search for potential biomarkers of sensitivity. However, the list of candidates is extensive and will continue to grow as additional functions of PARP-1 are discovered. Banking tumor biopsies from patients enrolled in PARP-1 clinical trials will greatly expedite the development of a panel of biomarkers, as will increased use of cancer genome sequencing and microarray technologies. Another challenge will be in identifying and overcoming mechanisms of resistance to PARP inhibition. For example, a second BRCA mutation or a deletion of the original mutation can cause reversion to HR-proficiency and resistance to PARP inhibitors in BRCA-mutated cancers (111). As the majority of clinical applications proposed here are theoretical or in pre-clinical development, associated mechanisms of resistance are entirely unknown, although development of such resistance is practically assured. Thirdly, many of the functions discussed here are effected by PARP-1 binding rather than enzymatic activity. Currently available PARP inhibitors act at the catalytic site of PARP-1, which does result in some degree of altered binding capacity via changes in autoPARylation status. However, treatment with PARP inhibitors may not effectively inhibit specific PARP-1 interactions, or may require different dosing. It will be important to study the various clinically available agents to determine if, and to what extent, binding domains are affected. Despite these obstacles, PARP inhibition is an extremely promising anticancer strategy and, as the first agents near completion of phase III trials, it will be exciting to see the magnitude of impact PARP inhibitors will have in clinical practice.

**Table 1:** **Summary of reported non-DNA repair functions of PARP-1 with potential clinical correlations**.

PARP-1 function	Effect	Model system studied	Clinical applicability of PARP inhibition
Binding histone acetyl-transferases p300/CBP	Co-activation of NF-κB (pro-inflammatory)	*In vitro* and *in vivo* HER2^+^ breast cancer cell lines	May inhibit cancer metastasis; cytotoxicity in HER2-positive breast cancer specifically (13, 21, 22)
Binding DNMT1	Enhances *Dnmt1* transcription, inhibits methyltransferase activity	*In vitro* mouse fibroblasts	May have activity in DNMT1-overexpressing colorectal, gastric, and hepatic carcinomas (85, 91, 92)
Binding pERK2	Promotes target gene transcription	*In vitro* endothelial cells	May inhibit cancer growth and metastasis (58)
Binding CHFR	Prophase arrest, resistance to microtubule inhibitors	*In vitro* gastric carcinoma cell lines	Re-sensitizes CHFR-expressing cancers to microtubule inhibitor therapy (75)
Downregulation of MKP-1 and inhibition of Akt	Activation of JNK	*In vitro* hepatocytes	May have activity in tumors with high JNK activity (46, 47)
AutoPARylation	Activation of NF-κB (pro-inflammatory)	*In vitro* and *in vivo* HER2^+^ breast cancer cell lines	May inhibit cancer metastasis; cytotoxicity in HER2-positive breast cancer specifically (13)
Caspase-8 PARylation	Impaired apoptotic signaling	*In vitro* and *in vivo* pancreatic cancer cell lines	Sensitizes cancer cells to TRAIL therapy (37)
PARylation	ATP depletion, promotes necrosis and autophagy	Mouse and rat kidney and heart studies	Prevents cell death mediated toxicities of multiple chemotherapy agents (42, 43, 52)
PARylation of transcription regulators	Promotes transcription	*In vitro* thyroid cancer cell lines	Upregulates NaI symporter transcription leading to increased radio-iodine uptake in thyroid cancer (96)
Androgen receptor PARylation	Increases androgen receptor activity	*In vivo* and *ex vivo* prostate cancer cells	Sensitizes prostate cancer to androgen depletion, enhances effects of anti-androgen therapy, delays onset of resistance to anti-androgen therapy (110)
ETS and EWS-FLI PARylation	Promotes transcription of target genes	*In vivo* and *in vitro* prostate cancer and sarcoma cells	Cytotoxicity in ETS-prostate cancer and EWS-FLI Ewing’s sarcoma specifically (14, 15)
Vimentin promoter PARylation	Promotes transcription	*In vitro* melanoma cells and *in vivo* melanoma model	Inhibits cancer metastasis (63)
Interaction with VEGF, SDC-4, PECAM1/CD31, HIF promoters	Promotes transcription	*In vitro* endothelial cells	Inhibits tumor angiogenesis (58–62)

## Author Contributions

Alice N. Weaver and Eddy S. Yang conceptualized the topic. Alice N. Weaver conducted the literature review and wrote the article. Eddy S. Yang critically revised the article and provided guidance and supervision.

## Conflict of Interest Statement

The authors declare that the research was conducted in the absence of any commercial or financial relationships that could be construed as a potential conflict of interest.
